# Acute compartment syndrome due to skeletal muscle metastases from poorly differentiated upper gastrointestinal adenocarcinoma: a case report

**DOI:** 10.1186/s12957-025-03696-3

**Published:** 2025-02-14

**Authors:** Richard Gentry, Prince Mohan Anand, Ahmed I Kamal, Ahmad Saleh Alqassieh, Ammar Obaid Mahmood, Mesrop Ayrapetyan, Monther Saud Amer Altiti

**Affiliations:** 1 Edward Via College of Osteopathic Medicine (VCOM), 2265 Kraft Drive SW, Blacksburg, VA USA; 2 Mid-Carolinas Kidney Transplant Program, Medical University of South Carolina, 820 West Meeting St, Lancaster, SC USA; 3 Department of Medicine, Division of Nephrology, Medical University of South Carolina, 96 Jonathan Lucas Street, Charleston, SC USA; 4 Department of General Surgery, Medical University of South Carolina, 800 W Meeting St, Lancaster, SC United States; 5 York Pathology Associates, Medical University of South Carolina, 800 W Meeting St, Lancaster, SC USA

**Keywords:** Upper gastrointestinal adenocarcinoma, Compartment syndrome, Muscle metastasis, Case report

## Abstract

**Background:**

Acute compartment syndrome (ACS) is characterized by increased pressure within the fascial network of any muscle, leading to impaired circulation and potential myonecrosis. Very rarely, soft tissue infiltration by metastatic disease can cause localized swelling that increases intercompartmental pressures. We report an unusual case of invasive, poorly differentiated upper gastrointestinal adenocarcinoma presented by acute compartment syndrome of the lower extremity and subsequent acute kidney injury (AKI) caused by myonecrosis-induced cast nephropathy.

**Case Presentation:**

A 52-year-old male presented to the hospital with rapid onset unilateral right leg pain and tense edema accompanied by myonecrosis with no explicable etiology complicated by AKI. Surgical fasciotomy and subsequent muscle biopsy yielded poorly differentiated non-small cell adenocarcinoma. CT imaging identified diffuse adenopathy along with abnormal thickening of the distal esophagus, gastroesophageal (GE) junction, and gastric cardia. Further investigation via upper esophagogastroduodenoscopy (EGD) revealed an exophytic mass in the distal esophagus extending into the stomach. This lesion was confirmed via biopsy as primary invasive poorly differentiated upper gastrointestinal (UGI) adenocarcinoma.

**Conclusion:**

This case highlights the need for clinicians to implement high-risk screening for UGI cancers and consider skeletal muscle metastasis as a cause of nontraumatic ACS. It emphasizes the importance of interdisciplinary collaboration in managing such complex cases and the role of timely surgical and oncological intervention in preventing long-term complications of ACS. Furthermore, it highlights the potential use of more efficient and specific MR imaging techniques to diagnose ambiguous cases of ACS.

## Background

Compartment syndrome is characterized by fascial compartment pressures surpassing perfusion pressure, resulting in cellular anoxia and potentially irreversible neuromuscular damage [1, 2]. ACS most often occurs in the lower extremities following trauma-related injuries such as fractures, penetrating wounds, or circumferential burns. The earliest and most reliable sign of ACS is pain out of proportion to physical exam that is exacerbated by passive stretching of the muscle [3]. Complications include myonecrosis, AKI, renal failure, and fatal cardiac arrythmias due to metabolic acidosis and hyperkalemia [4].

Esophageal adenocarcinoma (EAC) and gastric adenocarcinoma (GA) represent roughly 2.5% of all diagnosed cancers in the United States. EAC is proposed to originate from chronic Gastroesophageal Reflux Disease (GERD) leading to erosive esophagitis and intestinal metaplasia of the distal esophagus. Patients may present with dysphagia; however, more distal esophageal cancers closer to the GE junction, such as the one in this case, can present as isolated iron-deficiency anemia. During endoscopy, EAC can appear as a stricture, mass, ulceration, or subtle irregularity in the mucosa [5]. EAC most commonly metastasizes to regional lymph nodes; however, there have been rare cases where skeletal muscle metastasis has been described.

High body mass index, GERD, and smoking are all associated with an increased risk of adenocarcinomas of the distal esophagus, proximal stomach, and GE junction. Unfortunately, in GA most patients begin to have symptoms of abdominal pain, anorexia, and early satiety when the cancer has already reached an advanced stage [6, 7]. GA can resemble gastric ulcers with irregular borders, as well as clubbing, fusion, or amputation of radiating folds and nodularity of adjacent mucosa [8]. GA most commonly metastasizes to the liver, but there have been cases of skeletal muscle metastases. From a prognostic standpoint, the 5-year relative survival rate for localized EAC and GA is around 50%, whereas advanced disseminated EAC and GA with distant metastasis being as low as 0–6% [6, 9].

During an ACS, muscle necrosis and the disruption in cellular structure allow leakage of myoglobin into the bloodstream [10]. Myoglobin is filtered into the proximal tubule of the kidney where heme oxygenase-1 catalyzes the breakdown of heme to iron, carbon monoxide, and biliverdin [11]. Iron can combine with hydrogen peroxide to create ferric (Fe^3+^) iron, which increases hydroxyl ion concentrations via the iron redox cycle resulting in formation of reactive oxygen species and peroxidation of tubular cell membrane lipids [12]. Thus, myoglobin directly causes injury to tubular epithelial cells and compounds the injury by vascular effects such as the release of local vasoconstrictors in the vasa recta and peritubular capillaries. This mechanism is how an ACS can lead to AKI and imminent renal failure if left untreated.

## Case Presentation

**Fig. 1:**
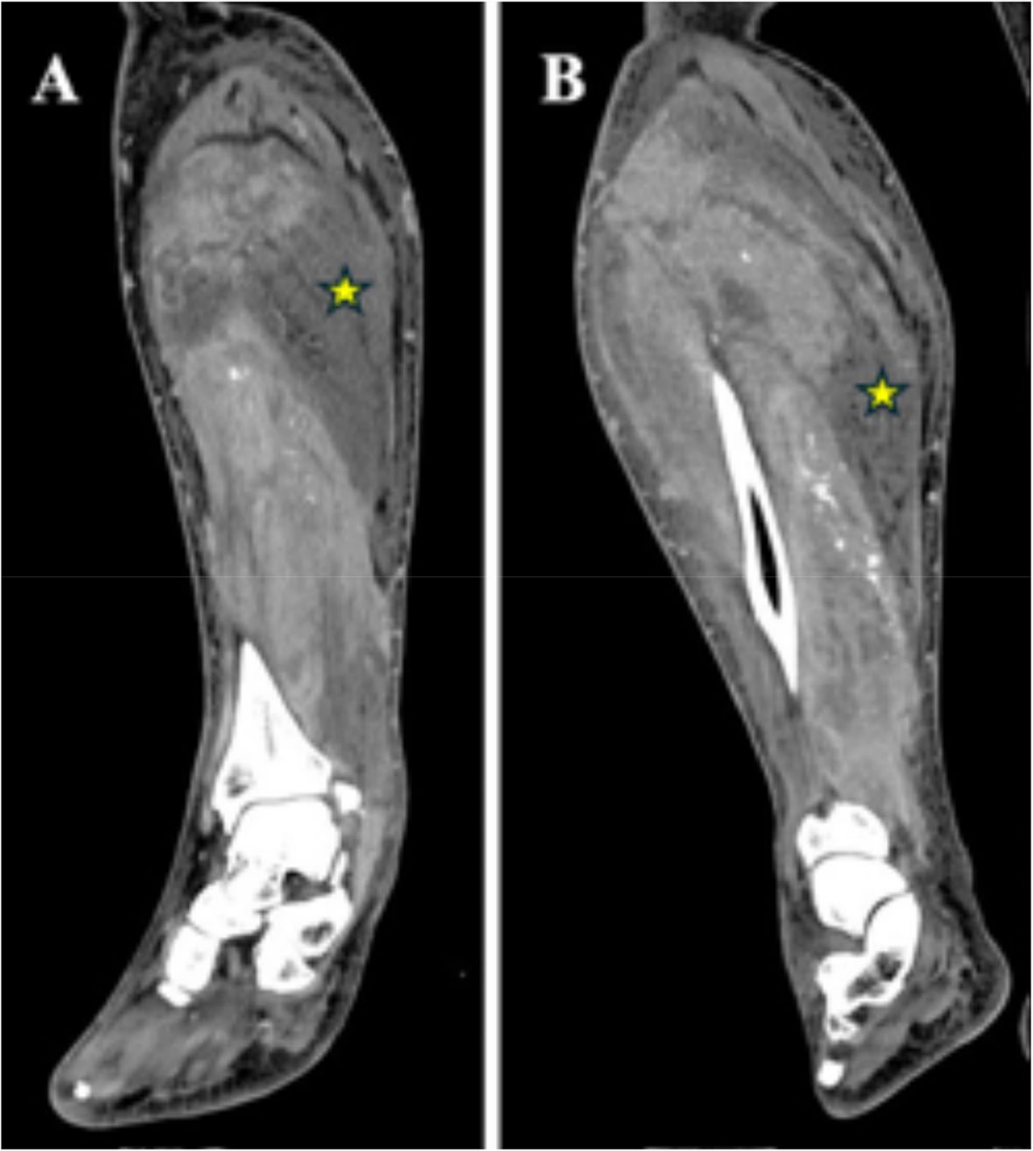
**A**, **B** Coronal CT images of the right lower extremity with IV contrast showing significant abnormal hypodensities within the right gastrocnemius muscle representing myonecrosis (stars)

A 51-year-old male was transferred to the hospital due to rapid unilateral right leg pain and swelling below the knee progressing over 24 h. Upon physical exam, the right lower leg was tense and painful to palpation and passive stretching. A CT scan was ordered and showed significant hypodensity within the muscles of the right lower extremity consistent with myonecrosis (Fig. 1). Pertinent labs indicated an elevated creatinine of 1.4 from a baseline of 0.6 mg/dl, and BUN of 40, indicating possible AKI. A creatinine kinase level of 6,200 IU/L was present, yielding significant muscular breakdown. Finally, a Complete Blood Count (CBC) showed significant anemia with decreased RBCs, hemoglobin, and hematocrit. 

The patient was immediately taken to the operating room, and a double-incision decompressive fasciotomy of the right lower extremity was performed, releasing all four compartments. Significant ischemic changes were appreciated in the gastrocnemius muscle, and a biopsy was taken for further pathological investigation. The incisions were left open with appropriate wound dressing, and no operative complications were noted. The leg was viable postoperatively.

**Fig. 2:**
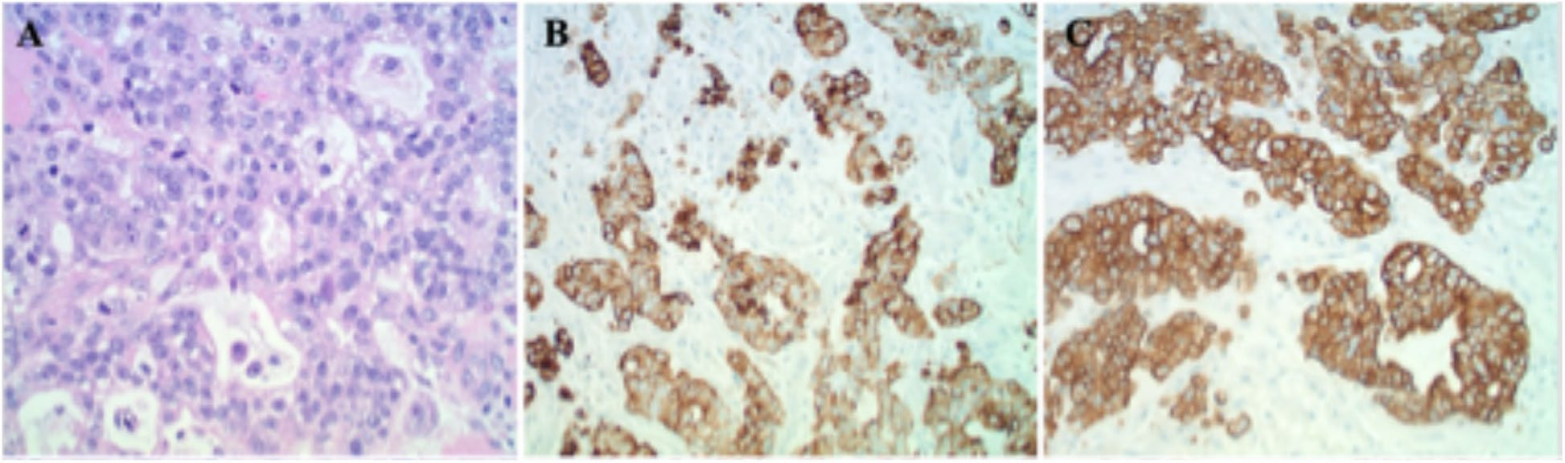
Biopsy of skeletal muscle excised during surgical fasciotomy (**A**) Hematoxylin and eosin staining at 200x magnification showing atypical poorly differentiated glandular cells with hyperchromatic nuclei, prominent nucleoli, and cystic formations. **B** CK7 immunohistochemistry staining of the same muscle biopsy at 100x magnification, this marker is expressed in adenocarcinoma of lung, breast, thyroid, endometrium, cervix, ovary, salivary gland, upper GI tract (**C**) Pancytokeratin immunohistochemistry staining of muscle biopsy at 100x magnification

**Fig. 3:**
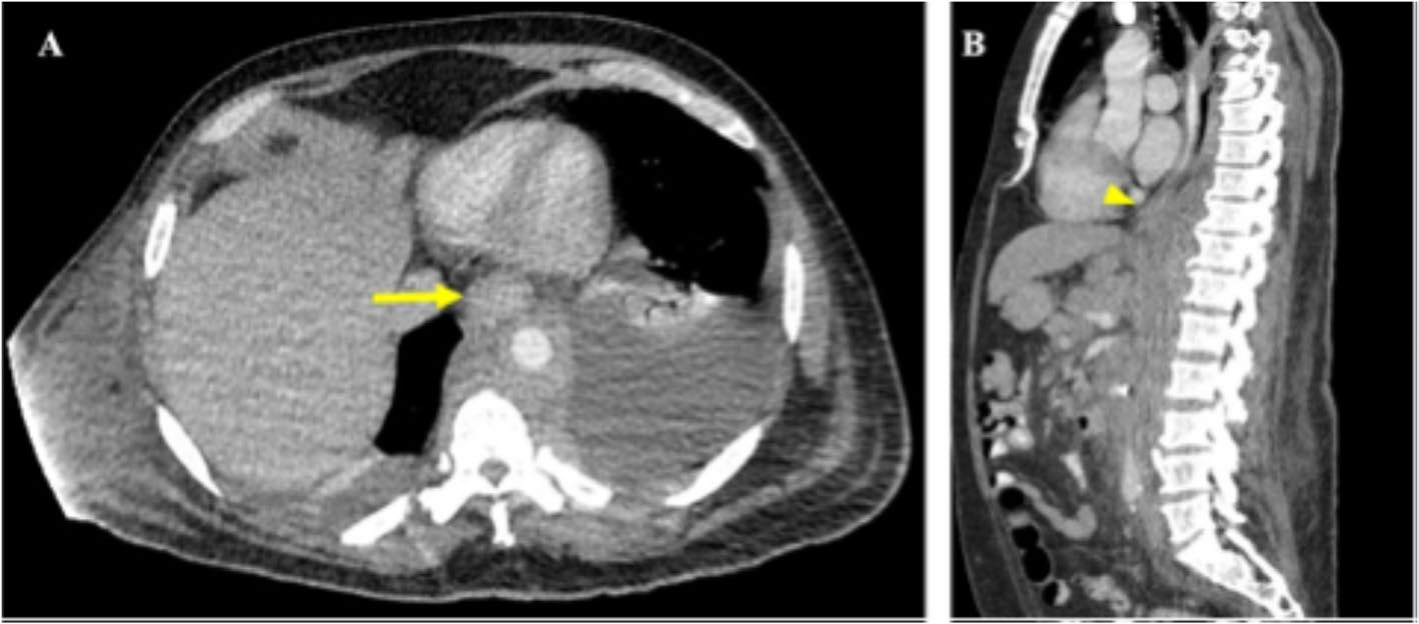
CT images of the chest/abdomen/pelvis with IV and oral contrast (**A**) Transverse section showing abnormal thickening of the distal esophagus (arrow). **B** Sagittal section showing abnormal thickening of the distal esophagus at the gastroesophageal junction (arrowhead)

Pathological analysis of the muscle biopsy yielded poorly differentiated non-small cell adenocarcinoma with mucinous features and focal signet ring morphology that stained positive for Pancytokeratin, CK7, and CDX2 on immunohistochemistry (Fig. 2). Both CK7 and CDX2 positivity together raise suspicion for UGI carcinoma. Additional workup included a CT with contrast scan of the chest, abdomen, and pelvis to determine if the primary malignancy was of gastrointestinal origin. This revealed extensive supraclavicular, mediastinal, gastrohepatic ligamentous, and retroperitoneal lymphadenopathy, as well as a marked thickening of the distal esophagus and GE junction extending into the gastric cardia (Fig. 3). A lymph node biopsy was then performed which paralleled the pathological findings of the right lower extremity muscle biopsy.

**Fig. 4:**
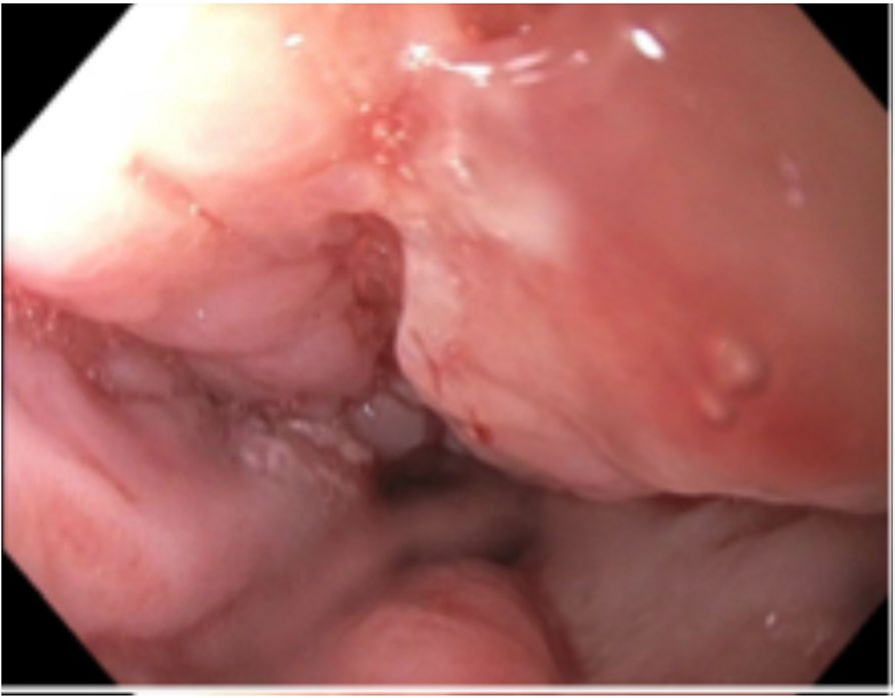
Upper esophagogastroduodenoscopy image of the distal esophagus near the gastroesophageal junction showing an asymmetric, lobulated mass with uneven, poorly defined borders protruding into the esophageal lumen, with areas of ulceration and surrounding tissue thickening

Gastroenterology was consulted, and a diagnostic EGD was performed, showing a large partially obstructing exophytic mass in the distal esophagus expanding into the GE junction through the cardia, fundus, and greater curvature of the stomach (Fig. 4). Biopsy from this mass yielded infiltrative poorly formed glands, compatible with invasive adenocarcinoma, matching the lymph node and muscle biopsy. The final pathological diagnosis was invasive poorly differentiated UGI adenocarcinoma. The primary UGI cancer metastasized to the lower extremity skeletal muscle, leading to soft tissue swelling and increased intercompartmental pressure, resulting in ACS, myonecrosis, and AKI due to myonecrosis-induced cast nephropathy. Unfortunately, there is no further information regarding treatment prognostics. After discovering the primary tumor, the patient opted to undergo hospice care and subsequently passed away shortly after.

## Discussion and conclusion

According to the American Cancer Society, recent data indicates an increase in gastric cardia adenocarcinoma and esophageal adenocarcinoma, particularly among younger adults due to diet, obesity, and environmental exposures. Given this rise in UGI adenocarcinoma incidence, there could be a steady increase in UGI adenocarcinoma-induced compartment syndrome such as the one described in this case. This highlights the need for early screening in high-risk patients, as well as improved diagnostic algorithms for nontraumatic ACS to potentially help decrease the mortality burden of UGI adenocarcinoma. Newer screening methods, such as a swallowable balloon device, can sample the distal esophagus and detect DNA methylation markers of esophageal metaplasia before carcinoma development with a sensitivity of 90.3% and a specificity of 91.7% [13]. Additionally, chromoendoscopy has shown increased diagnostic efficacy and detection of early gastric cancer and premalignant gastric conditions, with a sensitivity of 90% and specificity of 82% [14].

Although very rare, skeletal muscle metastasis of EAC has been reported. One study found that within a cohort of 205 patients diagnosed with esophageal carcinoma, four patients had skeletal muscle metastases [15]. In all these cases, pain due to skeletal muscle metastases was the first manifestation of systemic disease. This is significant to our case, given that our patient presented with ACS before any indication of metastatic UGI adenocarcinoma. In that same study, three out of the four patients’ palliation was obtained with the combination of external beam radiation, systemic chemotherapy, or surgical resection. Furthermore, a previous case reported a 76-year-old patient with a slowly progressive 2-week history of bilateral lower extremity swelling without ACS due to signet-ring cell GA metastases to skeletal muscle post-total gastrectomy. It was shown that the patient’s swelling significantly improved after the administration of chemotherapy [16]. This is significant because it shows that regardless of whether the tumor is of gastric or esophageal origin, UGI has the potential to metastasize to the muscle and lead to ACS. However, if we are more rapidly able to identify metastatic UGI adenocarcinoma as a possible cause for nontraumatic ACS, we can implement radiation, chemotherapy, or surgical resection to improve patients’ quality of life.

The main prognostic factors of ACS include injury severity, comorbidities, duration of cellular anoxia, and how rapidly a fasciotomy is performed. Studies have shown that if fasciotomy was performed within six hours of presentation, complete recovery of limb function can be achieved with little long-term complications [17]. However, when fasciotomy was delayed between six and twelve hours of presentation, normal limb function was regained in 68%; after twelve hours 8% regained normal function with increasing additional complications such as AKI and renal failure [18]. The patient’s rising creatinine and BUN were significant because it meant that the myonecrosis of the leg was releasing myoglobulin into the blood, causing AKI. If left untreated, his AKI could lead to renal failure and possibly long-term dialysis. If we can detect UGI adenocarcinoma earlier, it would be possible to prevent metastasis to the muscle, as well as decrease progression to ACS-induced AKI and potential renal failure.

**Fig. 5:**
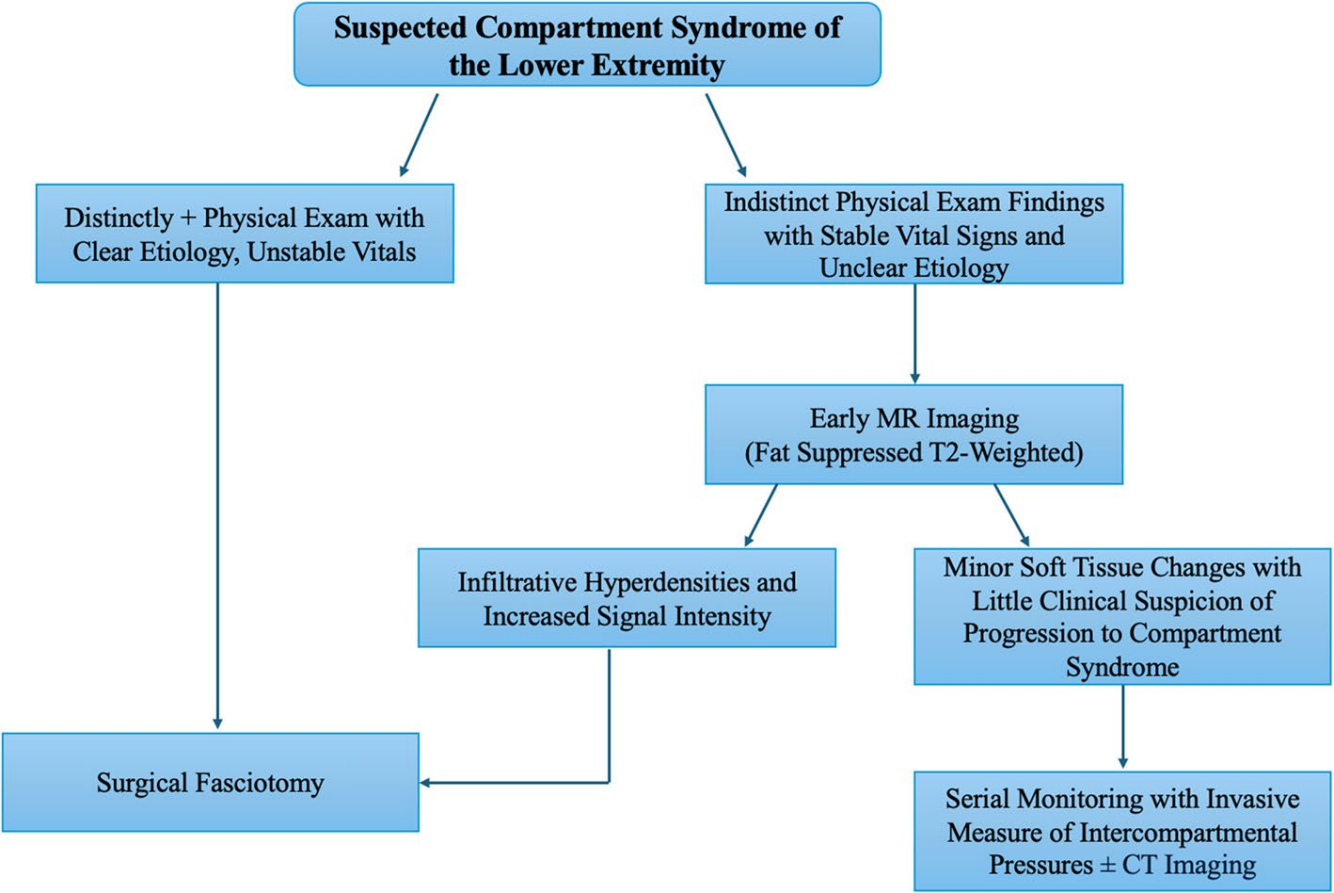
Proposed diagnostic and treatment algorithm for an ambiguous case of acute compartment syndrome. Distinctly positive physical exam including pain on passive extension accompanied by rapid, tense swelling, with any signs of sensory or motor deficits should undergo immediate surgical fasciotomy. This should also be the case in the presence of a clear etiology such as a long bone fracture in the lower extremity, or circumferential burns with unstable vitals such as tachycardia or hypotension. However, if the patient has absent physical exam findings mentioned earlier, with no signs of unstable vitals, and an unclear ideology, early fat suppressed T2-weighted MR imaging should be considered. If infiltrative hyperdensities and increased signal intensities on are noted, then a surgical fasciotomy should be performed as a prophylactic measure to prevent an acute compartment syndrome from occurring. MR imaging consistent with minor soft tissue changes, with little clinical suspicion of progression to a compartment syndrome should receive serial monitoring with invasive measurements of intercompartmental pressures with or without CT imaging to ensure neurovascular viability

Early MR imaging can provide a useful noninvasive tool to identify early atypical ACS prior to significant compartment pressure increases via needle. One study revealed that fat-suppressed T2-weighted MRI sequences are sensitive to early changes of muscle compartment edema shown as an infiltrative feather-like pattern of hyperintensity [19]. Early changes seen on MRI may allow surgeons to selectively split fascial spaces prophylactically to prevent ACS. Additionally, this approach simultaneously allows for identification of underlying soft tissue lesions such as malignant neoplasms within the skeletal muscle that can be addressed prior to, or during surgery. This could be a very useful tool in creating a possible treatment algorithm for ACS of unknown locations or etiologies (Fig. 5). One possible downfall of this method is stability of the patient given that an MRI of the lower extremity can take 30–60 min and may not be appropriate in a hyperacute setting. MRI could’ve been an alternative approach in our case giving us better visualization of soft tissue malignancy prior to fasciotomy.

There are significant cases of non-GI metastatic disease leading to ACS that provide greater insight into applying interdisciplinary treatment principles between surgery, oncology, and subspecialties such as nephrology to decrease mortality and improve quality of life. One case report describes metastatic melanoma to the flexor digitorum superficialis muscle causing a compartment syndrome of the forearm [20]. It was shown that with fasciotomy plus metastatic lesion excision, the patient made a favorable recovery. This is significant because if there is a metastatic growth within the muscle, excision of the metastasis as well as a fasciotomy may lead to a more favorable outcome than fasciotomy alone. Another case report showed a skeletal muscle-derived Non-Hodgkin Lymphoma presenting with compartment syndrome, and tumor lysis syndrome [21]. However, they proposed a muscle biopsy for pathological proof is crucial before or during the operation to create shared decision-making between physicians involved in treatment. This dilemma stems from the fact that systemic chemotherapy, and even radiation creates tissue swelling and inflammation that may exacerbate an already existing chronic compartment syndrome or create a new ACS. Additionally, they discussed the risk of tumor lysis syndrome that can present with both surgery and chemotherapy. Hyperkalemia, hyperuricemia, and hyperphosphatemia from tumor lysis syndrome can further compound AKI from an ACS and lead to an even more rapid kidney failure. This puts an emphasis on careful monitoring of kidney function through urine output and creatinine levels. Overall, this case details the necessary multidisciplinary treatment of metastatic-induced compartment syndrome between surgery, oncology, and subspecialties such as nephrology.

To our knowledge, there are no previously documented cases ACS due to skeletal muscle metastases of UGI adenocarcinoma. However, a steady increase in UGI adenocarcinoma has the potential to present a new diagnostic challenge for surgical oncologists managing patients with nontraumatic ACS. It is important to consider that lower extremity pain and edema may be the first presenting sign of highly advanced metastatic adenocarcinoma. However, we understand that the conclusions in the manuscript are based on a single case, which limits the generalizability. The reason this presentation may not have been previously reported is because of the short life expectancy of advanced metastatic UGI adenocarcinoma. Overall, our goal is to help clinicians faced with similar situations in the future come up with a good diagnostic approach to better outcomes for the patient. Future directions could empower more efficient and specific imaging techniques to diagnose an ambiguous ACS prior to life-threatening complications.

## Data Availability

No datasets were generated or analysed during the current study.

## Abbreviations

ACS: Acute compartment syndrome
EAC: Esophageal adenocarcinoma
GA: Gastric adenocarcinoma
AKI: Acute kidney injury
GE: Gastroesophageal
